# A Screen for Small Molecules to Target *Candida albicans* Biofilms

**DOI:** 10.3390/jof7010009

**Published:** 2020-12-27

**Authors:** Matthew B. Lohse, Craig L. Ennis, Nairi Hartooni, Alexander D. Johnson, Clarissa J. Nobile

**Affiliations:** 1 Department of Microbiology and Immunology, University of California—San Francisco, San Francisco, CA 94158, USA; matthew.lohse@ucsf.edu (M.B.L.); Nairi.Hartooni@ucsf.edu (N.H.); 2 Department of Biology, BioSynesis, Inc., San Francisco, CA 94114, USA; 3 Quantitative and Systems Biology Graduate Program, University of California—Merced, Merced, CA 95343, USA; cennis@ucmerced.edu; 4 Department of Molecular and Cell Biology, School of Natural Sciences, University of California—Merced, Merced, CA 95343, USA; 5 Department of Biochemistry and Biophysics, University of California—San Francisco, San Francisco, CA 94158, USA; 6 Health Sciences Research Institute, University of California—Merced, Merced, CA 95343, USA

**Keywords:** high-throughput screens, biofilms, biofilm inhibition, biofilm disruption, *Candida albicans*, antimicrobial resistance, therapeutics, Chembridge Small Molecule Diversity library

## Abstract

The human fungal pathogen *Candida albicans* can form biofilms on biotic and abiotic surfaces, which are inherently resistant to antifungal drugs. We screened the Chembridge Small Molecule Diversity library containing 30,000 “drug-like” small molecules and identified 45 compounds that inhibited biofilm formation. These 45 compounds were then tested for their abilities to disrupt mature biofilms and for combinatorial interactions with fluconazole, amphotericin B, and caspofungin, the three antifungal drugs most commonly prescribed to treat *Candida* infections. In the end, we identified one compound that moderately disrupted biofilm formation on its own and four compounds that moderately inhibited biofilm formation and/or moderately disrupted mature biofilms only in combination with either caspofungin or fluconazole. No combinatorial interactions were observed between the compounds and amphotericin B. As members of a diversity library, the identified compounds contain “drug-like” chemical backbones, thus even seemingly “weak hits” could represent promising chemical starting points for the development and the optimization of new classes of therapeutics designed to target *Candida* biofilms.

## 1. Introduction

*Candida albicans* is a normal commensal of the human microbiota that asymptomatically colonizes the skin, the mouth, and the gastrointestinal tract of healthy humans [1,2,3,4]. *C. albicans* is also one of the most common fungal pathogens of humans, typically causing superficial mucosal infections in healthy individuals [1,5,6,7,8,9,10,11]. When a host’s immune system is compromised (e.g., in patients with AIDS), *C. albicans* can give rise to disseminated bloodstream infections with mortality rates exceeding 40% [1,12,13,14,15].

A notable virulence trait of *C. albicans* is its ability to form biofilms, multilayered, structured communities of cells that can grow on biotic and abiotic surfaces, such as mucosal surfaces and implanted medical devices (e.g., catheters, dentures, and heart valves) [1,2,10,16,17,18,19,20,21]. These biofilms are often resistant to antifungal drugs at concentrations that are normally effective against planktonic (free-floating) cells [20,21,22,23,24,25]. The drug-resistant nature of *C. albicans* biofilms frequently makes removal of biofilm-infected medical devices the only effective option to mitigate a biofilm-based infection, which can be especially problematic if patients are already critically ill or when device removal requires surgical procedures (e.g., heart valve or prosthetic replacement) [20,26,27]. Since there are no biofilm-specific therapeutics available on the market today and only three major classes of antifungal drugs used to treat fungal infections in humans, the development of new therapeutics effective against *C. albicans* biofilms is an important and unmet medical need. 

The search for new antibiofilm therapeutics has encompassed a wide range of approaches, many of which focus on compounds that have combinatorial effects with known antifungal drugs rather than (or in addition to) compounds that affect *Candida* biofilms by themselves [28,29,30,31,32,33,34,35,36,37,38,39,40,41,42,43]. One approach has focused on screening libraries of existing drugs and/or pharmacologically active compounds that would make promising candidates for repurposing [38,39,40,43,44]. A second approach has focused on targeted classes of compounds (e.g., compounds with known effects on signaling pathways [28,29], compounds with known effects on cell–cell communication [34], and secreted aspartyl protease inhibitors [42]) that influence specific aspects of *Candida* biology. In addition, compounds that might affect *Candida* that are produced by other organisms (e.g., antimicrobial peptides [35] and chemicals produced by plants [36,37]) are also included in this targeted approach. A third approach has focused on screening large, chemically diverse compound libraries to identify pharmacophores that inhibit and/or disrupt biofilms through novel mechanisms [41,45]. Examples of this third approach taken to identify compounds with effects against *C. albicans* biofilms include a screen of a 20,000 compound Chembridge NOVACore library [45] and a screen of a 120,000 compound National Institutes of Health Molecular Libraries Small Molecule Repository library [41]. Here, we report a screen of a 30,000 compound Chembridge Small Molecule Diversity library (a library which, we note, has few compounds that overlap with the NOVACore library from the same commercial vendor) for the ability of the compounds to inhibit biofilm formation and/or disrupt mature biofilms by themselves or in combination with the known antifungal drugs fluconazole, amphotericin B, and caspofungin.

## 2. Materials and Methods

### 2.1. Media and Strains

Media were prepared in accordance with previously reported biofilm protocols [46,47]. Yeast extract peptone dextrose (YEPD) liquid media contains 2% Bacto^TM^ peptone (Difco #211677 (Becton, Dickinson and Company, Franklin Lakes, NJ, USA)), 2% dextrose, and 1% yeast extract (Difco #212750 (Becton, Dickinson and Company, Franklin Lakes, NJ, USA)). YEPD plates also contain 2% agar. Biofilm assays were performed in Roswell Park Memorial Institute (RPMI)-1640 media (containing L-glutamine and lacking sodium biocarbonate, MP Biomedicals #0910601 (MP Biomedicals, Santa Ana, CA, USA)) supplemented with 34.5 g/L 3-(*N*-morpholino)propanesulfonic acid (MOPS) (Sigma #M3183 (Sigma Aldrich, St. Louis, MO, USA)) and adjusted to pH 7.0 with sodium hydroxide before sterilizing using a 0.22 µm filter. All biofilm assays used the previously reported SC5314-derived strain SN425, a commonly used prototrophic **a**/α *C. albicans* standard strain, which was created by introducing *HIS1, LEU2*, and *ARG4* markers back into the SN152 **a**/α *his1 leu2 arg4* strain [48]. Cells were recovered from glycerol stocks for two days at 30 °C on yeast extract peptone dextrose (YEPD) plates. Overnight cultures for assays were grown approximately 16 h at 30 °C in YEPD media.

### 2.2. Reagents

The Chembridge Small Molecule Diversity library, which consists of 30,000 “drug-like” compounds, including diverse and target-directed compounds, was obtained by the University of California – San Francisco’s (UCSF’s) Small Molecule Discovery Center (SMDC) from commercial vendors and proprietary sources. Stocks of candidate compounds (as well as the three positive control compounds (PC12, 2-[(1,5-dimethyl-1*H*-pyrazol-4-yl)methyl]-7-(4-isopropylbenzyl)-2,7-diazaspiro[4.5]decane, Chembridge Catalog #17159859; PC26, 7-(4-isopropylbenzyl)-2-(tetrahydro-2H-thiopyran-4-yl)-2,7-diazaspiro[4.5]decan-6-one, Chembridge Catalog #80527891; PC27, 7-(4-isopropylbenzyl)-2-[(2-methyl-5-pyrimidinyl)methyl]-2,7-diazaspiro[4.5]decan-6-one, Chembridge Catalog #61894700) from the Chembridge NOVACore library that were hits from another high-throughput biofilm screen of a different Chembridge compound library [45]) were obtained directly from Chembridge (http://www.hit2lead.com/index.asp) for follow-up testing. Working stocks of the compounds were made at 20 mM in dimethyl sulfoxide (DMSO).

### 2.3. Biofilm Assays

The adherence inhibition, the sustained inhibition, and the disruption optical density biofilm assays followed previously reported 384-well format standard protocols [46,47,49,50]. In brief, for the biofilm inhibition assays, compounds were added during the 90 min adherence step (for the adherence and the sustained inhibition optical density biofilm assays) and/or at the 24 h growth step (for the sustained inhibition optical density biofilm assay). For the disruption optical density biofilm assay, a biofilm was grown for 24 h, after which the biofilm was incubated for an additional 24 h in the presence of the compound of interest. At the end of each assay, the media were removed from each well, and the OD_600_ of each well was measured using a Tecan Infinite M1000 Pro or a Tecan M200 plate reader (Tecan Group Ltd., Männedorf, Switzerland), taking the average of five reads per well.

The high-throughput adherence inhibition optical density biofilm assay screen of the Chembridge Small Molecule Diversity library was robotically conducted at UCSF’s SMDC. Compounds were included at a final concentration of 10 µM per well in 384-well plates in the high-throughput adherence inhibition optical density biofilm assay. Compounds were included at a final concentration of 40 µM per well in 384-well plates in the sustained inhibition optical density biofilm and the disruption optical density biofilm assays. Candidate compounds were tested at 12.5 µM in the combination sustained inhibition optical density biofilm and the disruption optical density biofilm assays. In line with previously reported studies [42,43], the combination sustained inhibition optical density biofilm assays used 1 µg/mL amphotericin B, 0.125 µg/mL caspofungin, or 256 µg/mL fluconazole. The combination disruption optical density biofilm assays used 2 µg/mL amphotericin B, 0.5 µg/mL caspofungin, or 256 µg/mL fluconazole. These amphotericin B, caspofungin, and fluconazole concentrations were chosen to be close to but below the effective concentrations (as measured by OD_600_) in the respective assays in order to leave a dynamic range for observing any combinatorial interactions. The sensitivity of SN425 to the antifungal drugs amphotericin B, caspofungin, and fluconazole in the sustained inhibition optical density biofilm and the disruption optical density biofilm assays is included in File S3.

### 2.4. Candidate Compound Selection

Candidate compounds based on the results of the adherence inhibition optical density biofilm assay high throughput screen of the 30,000 compound Chembridge Small Molecule Diversity library were selected as follows. Separate lists of candidate compounds were developed for those compounds with an absorbance at least two standard deviations below that of the DMSO only controls and for those compounds with a B-score of less than −4 [51,52]. Other factors were also included in our selection criteria prioritizations, such as the selection of compounds with most favorable chemistries for optimizations as well as the selection of compounds that are available for purchase from Chembridge (http://www.hit2lead.com/index.asp) (some compounds became unavailable for commercial purchase during this study). In total, we selected 64 compounds, 28 from the standard deviation list and 36 from the B-score list. Nineteen of these compounds were on both lists for a total of 45 candidate compounds (File S2). We selected three additional compounds available from Chembridge (PC12, Chembridge Catalog #17159859; PC26, Chembridge Catalog #80527891; PC27, Chembridge Catalog #61894700) that were not in the Chembridge Small Molecule Diversity library but were previously reported by Pierce and colleagues to inhibit biofilm formation in a screen of a different Chembridge libraries (the Chembridge NOVACore library) [45] to serve as positive controls. Data from our adherence inhibition optical density biofilm assay screen of the 30,000 compound Chembridge Small Molecule Diversity library can be found in File S1. A list of the 45 selected candidate compounds can be found in File S2.

### 2.5. Statistical Analysis and “Hit” Calling for the Biofilm Assays

Statistical analyses and “hit” calling for the Biofilm Assays followed previously reported protocols [42,43]. For the stand-alone sustained inhibition and disruption optical density biofilm assays, individual repeats of candidate compounds (and controls) were performed in groups of eight wells. Between two and four repeats (16–32 total wells) were performed for each candidate compound. Each plate had seven sets of control wells (56 total wells) containing equivalent volumes of DMSO to the experimental wells spread throughout the plate to reduce positional effects. For each experimental set of eight wells, significance was evaluated relative to all of the control wells from the same plate using Welch’s *t*-tests (two-tailed, assuming unequal variance). In order to correct for the multiple comparisons performed, we then applied the Bonferroni correction with α = 0.05. All of the comparisons for a given type of assay (e.g., all of the stand-alone sustained inhibition optical density biofilm assays) were pooled for the multiple comparisons correction step, giving a number of hypotheses, m, of 146 for the sustained inhibition optical density biofilm assay and of 105 for the disruption optical density biofilm assay (for final thresholds of 3.42 × 10^−4^ and 4.76 × 10^−4^, respectively). We then determined whether each experimental repeat (1) had an average absorbance less than the average of the control wells and (2) was significant after the multiple comparisons correction. To be considered a validated “hit”, a compound had to satisfy both of these criteria.

For the combination sustained inhibition and disruption optical density biofilm assays, compounds (and controls) were again tested in groups of eight wells, and two distinct groups of controls were included on each plate. The first set of controls were wells where the candidate compound but no known antifungal drug was included. The second set of controls were wells where the antifungal drug but no candidate compound was included. In both cases, we used the same concentration of candidate compound or antifungal drug as was used in the experimental wells. Controls were included for all candidate compounds and antifungal drugs being tested on a given plate. In general, a single set of eight wells was included for each experimental or control condition on a given plate. Statistical analysis was performed using Welch’s *t*-test and the Bonferroni correction as described above with the following modifications. Each experimental condition was compared to both the relevant antifungal drug control and the relevant candidate control (e.g., a compound CB01 plus caspofungin experiment was compared to the CB01 only control and the caspofungin only control from the same plate). All of the same comparisons for a given assay (e.g., all of the antifungal drug comparisons for the combination sustained inhibition optical density biofilm assay) were pooled for the multiple comparisons correction step, giving a number of hypotheses, m, of 144 for both the antifungal drug and the candidate compound comparisons in both the sustained inhibition and the disruption optical density biofilm assays (for a final threshold of 3.47 × 10^−4^). To be considered a validated combination hit, a given experimental condition had to have (1) an average absorbance less than the averages of both sets of relevant control wells and (2) remain significant after the multiple comparisons correction for both sets of comparisons.

Data and statistics for the stand-alone and the combination biofilm assays are compiled in File S3. The chemical properties of the “hit” compounds (including molecular weights, polar surface area, logP, logSW, the number of rotatable bonds, and the numbers of H-bond acceptors and donors) that were available at the ChemBridge Online Chemical Store (www.hit2lead.com) are also included in File S3.

## 3. Results

**Figure 1:**
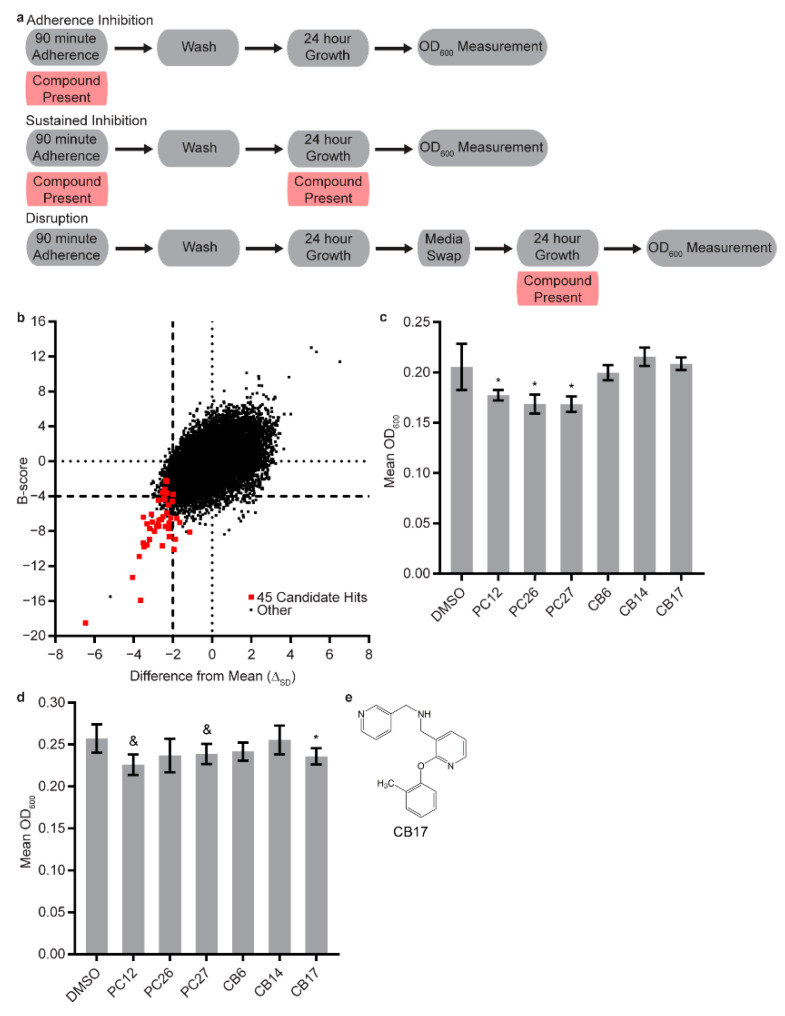
Screen of the Chembridge 30,000 “drug-like” member library for compounds with the ability to inhibit *C. albicans* biofilm formation. (**a**) Overview of the adherence inhibition, the sustained inhibition, and the disruption optical density biofilm assays. (**b**) Comparisons of the differences from the mean (in units of standard deviation, *x*-axis) and the B-score (*y*-axis) for the entire library screened at a concentration of 10 µM in the adherence inhibition optical density biofilm assay. The 45 candidate hits that were pursued further are indicated in red, and all other compounds are indicated in black. (**c**,**d**) Statistically significant hits, positive controls, and additional selected candidates from the (**c**) stand-alone sustained inhibition optical density biofilm assay and the (**d**) stand-alone disruption optical density biofilm assay; compounds were included at concentrations of 40 µM. In both panels, the mean OD_600_ readings with standard deviations are shown. Significant differences from the DMSO solvent control, as determined by Welch’s *t*-test (two-tailed, assuming unequal variance) with the Bonferroni correction, are indicated for α = 0.05 (*) or mixed results (&). In the cases of PC12 (2-[(1,5-dimethyl-1*H*-pyrazol-4-yl)methyl]-7-(4-isopropylbenzyl)-2,7-diazaspiro[4.5]decane, Chembridge Catalog #17159859) and PC27 (7-(4-isopropylbenzyl)-2-[(2-methyl-5-pyrimidinyl)methyl]-2,7-diazaspiro[4.5]decan-6-one, Chembridge Catalog #61894700) in the disruption optical density biofilm assay, only one of the two repeats performed met the significance threshold. Data within a chart were taken from the same plate. (**e**) Structure of compound CB17 (1-[2-(2-methylphenoxy)-3-pyridinyl]-*N*-(3-pyridinylmethyl)methanamine, Chembridge Catalog #80338143) disrupted mature *C. albicans* biofilms on its own at a concentration of 40 µM.

The Chembridge Small Molecule Diversity library of 30,000 “drug-like” compounds covering a wide range of chemical scaffolds, diverse chemical backbones, chemotypes, and pharmacophores was robotically screened for compounds that inhibit *C. albicans* biofilm formation. This screen used the adherence inhibition optical density biofilm assay [46,47] (Figure 1a), where the compound of interest was added during the 90 min initial step of biofilm formation and then washed out (along with unadhered cells). The biofilm was then allowed to develop for 24 h in the absence of the compound. In total, 45 candidate compounds were then selected for further evaluation in secondary assays (Figure 1b, Files S1 and S2).

The 45 candidate Chembridge compounds (as well as the three positive control Chembridge compounds previously reported to inhibit biofilm formation that were not present in the 30,000 compound Small Molecule Diversity library [45]) were then evaluated for antibiofilm activity in the sustained inhibition optical density biofilm assay and the disruption optical density biofilm assay [46,47]. In the sustained inhibition optical density biofilm Assay, the compounds were added to the media during both the 90 min adherence step and the 24 h growth step of biofilm formation (Figure 1a). In the disruption optical density biofilm assay, a biofilm was grown for 24 h, after which the biofilm was incubated for an additional 24 h in the presence of the compound (Figure 1a). Other than the three positive controls (PC12, 2-[(1,5-dimethyl-1H-pyrazol-4-yl)methyl]-7-(4-isopropylbenzyl)-2,7-diazaspiro[4.5]decane, Chembridge Catalog #17159859; PC26, 7-(4-isopropylbenzyl)-2-(tetrahydro-2*H*-thiopyran-4-yl)-2,7-diazaspiro[4.5]decan-6-one, Chembridge Catalog #80527891; PC27, 7-(4-isopropylbenzyl)-2-[(2-methyl-5-pyrimidinyl)methyl]-2,7-diazaspiro[4.5]decan-6-one, Chembridge Catalog #61894700) [45], none of the compounds tested inhibited biofilm formation throughout the duration of biofilm development (Figure 1c and Figure S1a). We do not fully understand why some compounds showed significant inhibition in the adherence inhibition optical density biofilm assay but not in the sustained inhibition optical density biofilm assay, but these different assays may be sensitive to different compound parameters such as solubility, stability, and pH dependence. Given the lack of a biofilm inhibition phenotype in the sustained inhibition optical density biofilm assay, we were surprised to find that one of the 45 compounds (CB17, 1-[2-(2-methylphenoxy)-3-pyridinyl]-*N*-(3-pyridinylmethyl)methanamine, Chembridge Catalog #80338143) disrupted mature *C. albicans* biofilms on its own at the same concentration (Figure 1d,e and Figure S1b). See File S3 for names and chemical properties of this compound.

**Figure 2:**
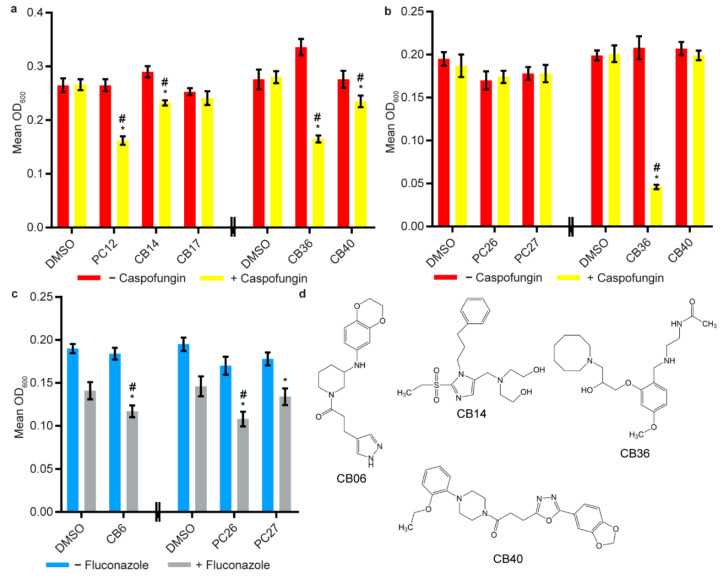
Combination screening of candidate compounds with the antifungal drugs caspofungin and fluconazole. (**a**) combination disruption optical density biofilm assay and (**b**) combination sustained inhibition optical density biofilm assay with caspofungin. For each compound, wells with caspofungin (+ caspofungin) are indicated in yellow, and wells without caspofungin (− caspofungin) are indicated in red. (**c**) Combination sustained inhibition optical density biofilm assay with fluconazole. For each compound, wells with fluconazole (+ fluconazole) are indicated in grey and wells without fluconazole − fluconazole) are indicated in blue. Mean OD_600_ readings with standard deviations are shown, significant differences from the compound without antifungal drug controls (e.g., PC12, − caspofungin), as determined by Welch’s *t*-test (two-tailed, assuming unequal variance) with the Bonferroni correction, are indicated for α = 0.05 (*). Significant differences from the antifungal drug without compound control (e.g., DMSO, + caspofungin), determined by the same statistical analysis, are indicated for α = 0.05 (#). Data from different plates are separated by two vertical lines on the *x*-axis, and DMSO solvent controls are shown for each plate. Candidate compounds were included at concentrations of 12.5 µM in each of these assays. (**d**) Structures of compounds CB06 (*N*-(2,3-dihydro-1,4-benzodioxin-6-yl)-1-[3-(1*H*-pyrazol-4-yl)propanoyl]-3-piperidinamine, Chembridge Catalog #22164746), CB14 (2,2′-({[2-(ethylsulfonyl)-1-(3-phenylpropyl)-1H-imidazol-5-yl]methyl}imino)diethanol, Chembridge Catalog #10068182), CB36 (*N*-[2-({2-[3-(1-azocanyl)-2-hydroxypropoxy]-4-methoxybenzyl}amino)ethyl]acetamide, Chembridge Catalog #29059737), and CB40 (1-{3-[5-(1,3-benzodioxol-5-yl)-1,3,4-oxadiazol-2-yl]propanoyl}-4-(2-ethoxyphenyl)piperazine, Chembridge Catalog #35558198) inhibited and/or disrupted *C. albicans* biofilms in combination with at least one of the known antifungal drugs tested.

Given the previous reports suggesting antibiofilm synergies between known antifungal drugs and certain drug classes, we next tested our initial 45 candidate compounds for their abilities to inhibit biofilm formation (using the sustained inhibition optical density biofilm assay and/or to disrupt mature biofilms (using the disruption optical density biofilm assay) when combined with sub-inhibitory concentrations of amphotericin B, caspofungin, or fluconazole (Figure 2 and Figures S2 and S3). Three compounds disrupted mature biofilms in the presence of caspofungin (CB14, 2,2′-({[2-(ethylsulfonyl)-1-(3-phenylpropyl)-1*H*-imidazol-5-yl]methyl}imino)diethanol, Chembridge Catalog #10068182; CB36, *N*-[2-({2-[3-(1-azocanyl)-2-hydroxypropoxy]-4-methoxybenzyl}amino)ethyl]acetamide, Chembridge Catalog #29059737; CB40, 1-{3-[5-(1,3-benzodioxol-5-yl)-1,3,4-oxadiazol-2-yl]propanoyl}-4-(2-ethoxyphenyl)piperazine, Chembridge Catalog #35558198) (Figure 2a and Figure S2a). One of these compounds (CB36) also inhibited biofilm formation in the presence of caspofungin (Figure 2b and Figure S3a). In addition, a fourth compound (CB06, *N*-(2,3-dihydro-1,4-benzodioxin-6-yl)-1-[3-(1*H*-pyrazol-4-yl)propanoyl]-3-piperidinamine, Chembridge Catalog #22164746) inhibited biofilm formation in the presence of fluconazole (Figure 2c and Figure S3b). As noted above, none of these compounds had effects on biofilms on their own in this assay. Chemical properties of these compounds can be found in File S3. We also note that the positive control compounds PC12, PC26, and PC27 all disrupted mature biofilms in the presence of caspofungin, PC12 disrupted mature biofilms in the presence of fluconazole, and PC26 inhibited biofilm formation in the presence of fluconazole (Figure 2 and Figures S2 and S3).

## 4. Discussion

Starting from an initial screen of a 30,000 compound diversity library and following standard high-throughput screening procedures for hit identification [53], we identified four compounds capable of inhibiting biofilm formation and/or disrupting mature biofilms in combination with caspofungin or fluconazole and a fifth compound capable of disrupting mature *C. albicans* biofilms on its own. As members of a diversity library, the identified compounds contain “drug-like” chemical backbones that represent promising chemical starting points for the development and the optimization of new classes of therapeutics designed to target *Candida* biofilms. For example, all compounds within this library have low molecular weights, low polar surface areas, and are predicted to be soluble and capable of crossing membranes. Given the distinct structures of our specific individual and combination hits, these compounds are likely to display broad ranges of biological activities and should provide multiple amenable opportunities for structural elaboration. Thus, even seemingly “weak” hits have the potential to become potent hits upon chemical optimizations [53,54,55]. Therefore, even compounds we identified with relatively minor yet significant antibiofilm effects on their own (e.g., CB17) have promise. In addition, our combination results indicate potent effects for certain compounds (e.g., CB06, CB14, CB36, CB40) in combination with fluconazole and caspofungin, suggesting that these compounds are a priority for future chemical optimizations.

In addition to identifying several promising antibiofilm compounds, our results illustrate the degree to which the experimental setup for biofilm formation can affect compound efficacy. One example is our identification of several compounds with efficacy in combination with known antifungal drugs, where the combined effect is dependent on the assay conditions. A second example is our identification of compounds that disrupt mature biofilms but that do not inhibit biofilm formation (either on their own or in combination with known antifungal drugs). Given these findings, drug efficacy testing that focuses solely on one aspect of biofilm formation (e.g., inhibition of initial biofilm formation) may overlook promising compounds that may be broadly effective against mature biofilms, and vice versa. Thus, multiple testing parameters of compounds against different stages of biofilm formation are useful in identifying the most promising compounds for therapeutic development.

## Supplementary Materials

The following are available online at https://www.mdpi.com/2309-608X/7/1/9/s1. File S1, Screen of the Chembridge 30,000 “drug-like” member library for compounds with the ability to inhibit *C. albicans* biofilm formation in the adherence inhibition optical density biofilm assay. Differences from the mean (in units of standard deviation) and the B-score for the entire library screened at a concentration of 10 µM are provided. File S2, Identities of the 45 candidate compounds selected based on the initial adherence inhibition optical density biofilm assay as well as the three positive controls. Differences from the mean (in units of standard deviation) and the B-score are indicated for these compounds. File S3, Compiled data and statistics from the stand-alone and combination sustained inhibition and disruption optical density biofilm assays. For each compound, the average OD_600_, average OD_600_ of relevant control(s), and value(s) for Welch’s *t*-test versus the relevant control(s) are provided. Whether the average OD_600_ was below the average OD_600_ of the relevant control(s) and whether the difference from the relevant control(s) remains significant following the Bonferroni Correction (α = 0.05) are indicated. The chemical properties of the “hit” compounds (including molecular weights, polar surface area, logP, logSW, the number of rotatable bonds, and the numbers of H-bond acceptors and donors) that were available at the ChemBridge Online Chemical Store (www.hit2lead.com) are also included. Figure S1, Additional results from the (**a**) stand-alone sustained inhibition and the (**b**) stand-alone disruption optical density biofilm assays. Mean OD_600_ readings with standard deviations are shown, significant differences from the DMSO solvent control, as determined by Welch’s *t*-test (two-tailed, assuming unequal variance) with the Bonferroni Correction, are indicated for α=0.05 (*) or mixed results (&). In the cases of CB36 and CB40 in the sustained inhibition optical density biofilm assay, only one of the two repeats performed met the significance threshold. In the case of CB40 in the disruption optical density biofilm assay, only two of the four repeats performed met the significance threshold. Data within a chart are all taken from the same plate on the same day. Figure S2, Additional results from the disruption optical density biofilm assay combination screening of candidate compounds with the antifungal agents caspofungin, fluconazole, and amphotericin B. Combination disruption biofilm assays with (**a**) caspofungin, (**b**) fluconazole, and (**c**) amphotericin B. In panel **a**, wells with caspofungin (+ caspofungin) are indicated in yellow and wells without caspofungin (− caspofungin) are indicated in red. In panel **b**, wells with fluconazole (+ fluconazole) are indicated in grey and wells without fluconazole (−fluconazole) are indicated in blue. In panel **c**, wells with amphotericin B (+ amphotericin B) are indicated in orange and wells without amphotericin B (− amphotericin B) are indicated in green. Mean OD_600_ readings with standard deviations are shown, significant differences from the compound without antifungal agent controls (e.g., CB6, − caspofungin), as determined by Welch’s *t*-test (two-tailed, assuming unequal variance) with the Bonferroni Correction, are indicated for α = 0.05 (*). Significant differences from the antifungal agent without compound control (e.g., DMSO, + caspofungin), determined by the same statistical testing, are indicated for α = 0.05 (#). Candidate compounds were included at a concentration of 12.5 µM. Data from different plates are separated by two vertical lines on the x-axis, DMSO solvent controls are shown for each plate. Figure S3, Additional results from the sustained inhibition optical density biofilm assay combination screening of candidate compounds with the antifungal agents caspofungin, fluconazole, and amphotericin B. Combination sustained inhibition assays with (**a**) caspofungin, (**b**) fluconazole, and (**c**) amphotericin B. In panel **a**, wells with caspofungin (+ caspofungin) are indicated in yellow and wells without caspofungin (− caspofungin) are indicated in red. In panel **b**, wells with fluconazole (+ fluconazole) are indicated in grey and wells without fluconazole (− fluconazole) are indicated in blue. In panel **c,** wells with amphotericin B (+ amphotericin B) are indicated in orange and wells without amphotericin B (− amphotericin B) are indicated in green. Mean OD_600_ readings with standard deviations are shown, significant differences from the compound without antifungal agent controls (e.g., CB6, − caspofungin), as determined by Welch’s *t*-test (two-tailed, assuming unequal variance) with the Bonferroni Correction, are indicated for α=0.05 (*). Significant differences from the antifungal agent without compound control (e.g. DMSO, + caspofungin), determined by the same statistical tests, are indicated for α=0.05 (#). Candidate compounds were included at a concentration of 12.5 µM. Data from different plates are separated by two vertical lines on the x-axis, DMSO solvent controls are shown for each plate.
