# Supplementary material for: A Screen for Small Molecules to Target Candida albicans Biofilms
Source: J Fungi (Basel). 2020 Dec 27;7(1):9. doi: 10.3390/jof7010009 (PMC7824004; doi:10.3390/jof7010009)
Supplement: Supplementary file 1 [file jof-07-00009-s001.zip › Supplemental Figure and File Legends.pdf]

Supplemental Figure and File Legends:

Figure S1. Additional results from the (a) stand-alone Sustained Inhibition and the (b) stand-alone Disruption Optical Density Biofilm Assays. Mean OD_{600} readings with standard deviations are shown, significant differences from the DMSO solvent control, as determined by Welch's t-test (two-tailed, assuming unequal variance) with the Bonferroni Correction, are indicated for $\alpha=0.05$ (*) or mixed results (&). In the cases of CB36 and CB40 in the Sustained Inhibition Optical Density Biofilm Assay, only one of the two repeats performed met the significance threshold. In the case of CB40 in the Disruption Optical Density Biofilm Assay, only two of the four repeats performed met the significance threshold. Data within a chart are all taken from the same plate on the same day.

Figure S2. Disruption Optical Density Biofilm Assay combination screening of candidate compounds with the antifungal drugs caspofungin, fluconazole, and amphotericin B. Combination disruption biofilm assays with (a) caspofungin, (b) fluconazole, and (c) amphotericin B. In panel a, wells with caspofungin (+ caspofungin) are indicated in yellow and wells without caspofungin (- caspofungin) are indicated in red. In panel b, wells with fluconazole (+ fluconazole) are indicated in grey and wells without fluconazole (-fluconazole) are indicated in blue. In panel c, wells with amphotericin B (+ amphotericin B) are indicated in orange and wells without amphotericin B (- amphotericin B) are indicated in green. Mean OD_{600} readings with standard deviations are shown, significant differences from the compound without antifungal drug controls (e.g. CB6, - caspofungin), as determined by Welch's t-test (two-tailed, assuming unequal variance) with the Bonferroni Correction, are indicated for $\alpha=0.05$ (*).

Significant differences from the antifungal drug without compound control (e.g. DMSO, + caspofungin), determined by the same statistical testing, are indicated for α=0.05 (#). Candidate compounds were included at concentrations of 12.5 µM. Data from different plates are separated by two vertical lines on the x-axis, DMSO solvent controls are shown for each plate.

Figure S3. Sustained Inhibition Optical Density Biofilm Assay combination screening of candidate compounds with the antifungal drugs caspofungin, fluconazole, and amphotericin B. Combination sustained inhibition assays with (a) caspofungin, (b) fluconazole, and (c) amphotericin B. In panel a, wells with caspofungin (+ caspofungin) are indicated in yellow and wells without caspofungin (- caspofungin) are indicated in red. In panel b, wells with fluconazole (+ fluconazole) are indicated in grey and wells without fluconazole (- fluconazole) are indicated in blue. In panel c, wells with amphotericin B (+ amphotericin B) are indicated in orange and wells without amphotericin B (- amphotericin B) are indicated in green. Mean OD_{600} readings with standard deviations are shown, significant differences from the compound without antifungal drug controls (e.g. CB6, - caspofungin), as determined by Welch's t-test (two-tailed, assuming unequal variance) with the Bonferroni Correction, are indicated for α=0.05 (*). Significant differences from the antifungal drug without compound control (e.g. DMSO, + caspofungin), determined by the same statistical tests, are indicated for α=0.05 (#). Candidate compounds were included at concentrations of 12.5 µM. Data from different plates are separated by two vertical lines on the x-axis, DMSO solvent controls are shown for each plate.

File S1. Screen of the Chembridge 30,000 "drug-like" member library for compounds with the ability to inhibit *C. albicans* biofilm formation in the Adherence Inhibition Optical Density

Biofilm Assay. Differences from the mean (in units of standard deviation) and the B-score for the entire library screened at a concentration of 10 µM are provided.

File S2. Identities of the 45 candidate compounds selected based on the initial Adherence Inhibition Optical Density Biofilm Assay as well as the three positive controls. Differences from the mean (in units of standard deviation) and the B-score are indicated for these compounds.

File S3. Compiled data and statistics from the Stand-alone and Combination Sustained Inhibition and Disruption Optical Density Biofilm Assays. For each compound, the average OD_{600}, average OD_{600} of relevant control(s), and value(s) for Welch's t-test versus the relevant control(s) are provided. Whether the average OD_{600} was below the average OD_{600} of the relevant control(s) and whether the difference from the relevant control(s) remains significant following the Bonferroni Correction (α=0.05) are indicated.
