# Supplementary figures and images for: A Screen for Small Molecules to Target Candida albicans Biofilms
Source: J Fungi (Basel). 2020 Dec 27;7(1):9. doi: 10.3390/jof7010009 (PMC7824004; doi:10.3390/jof7010009)

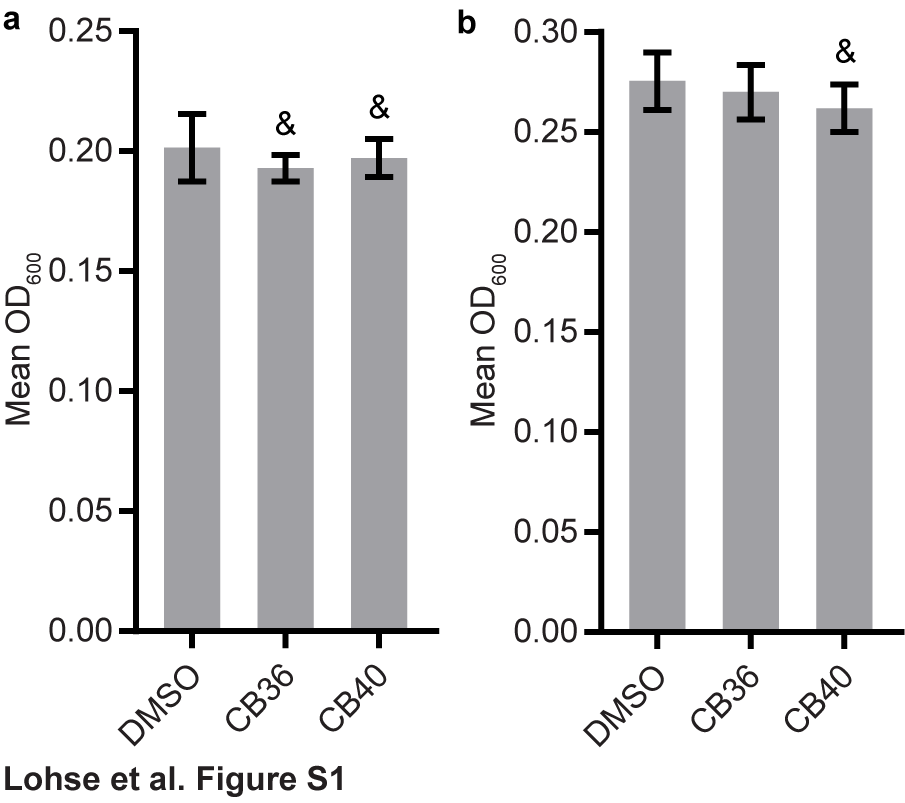

Supplement: Supplementary file 1 [file jof-07-00009-s001.zip › Lohse et al Figure S1.tif]

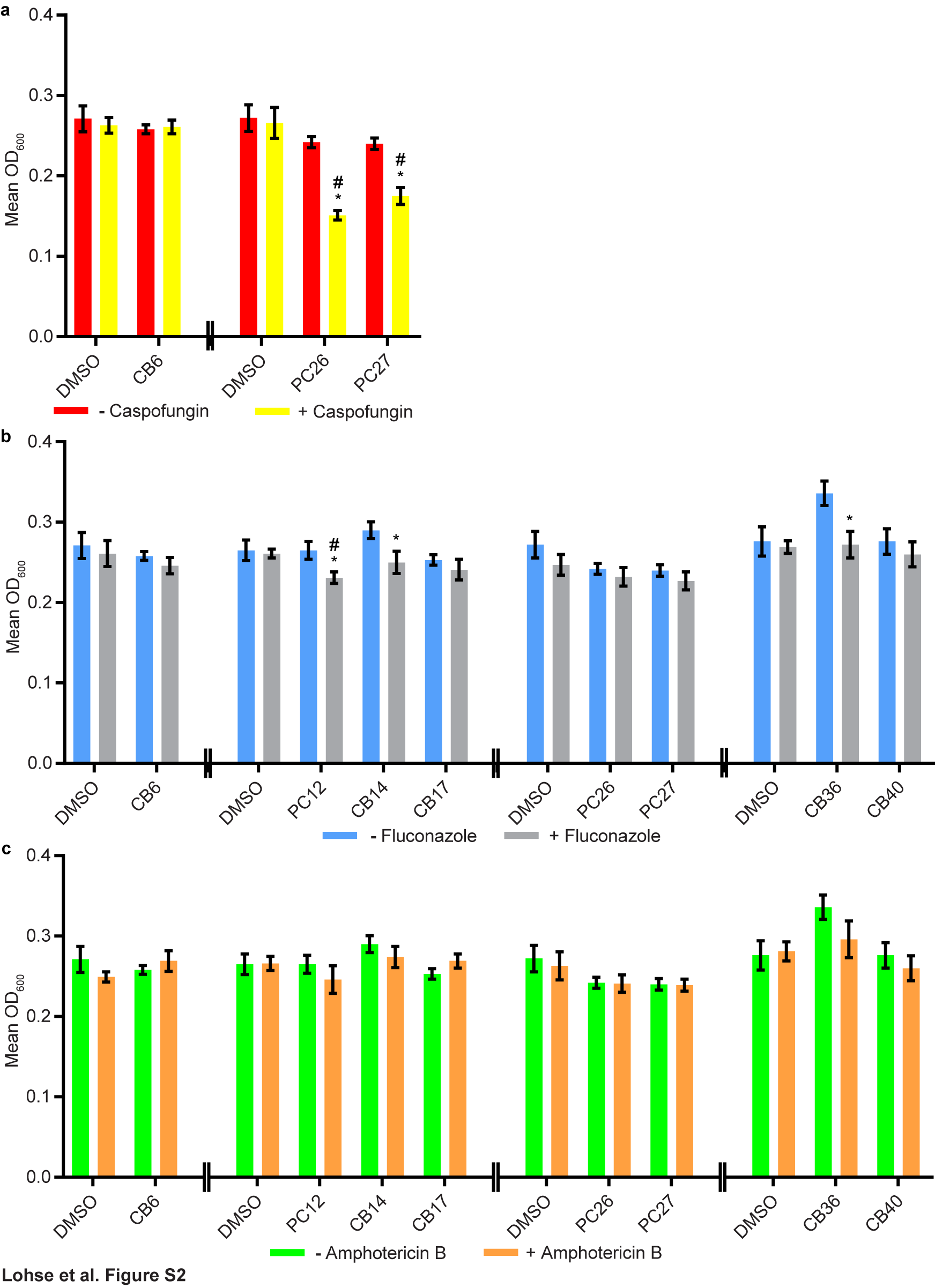

Supplement: Supplementary file 1 [file jof-07-00009-s001.zip › Lohse et al Figure S2.tif]

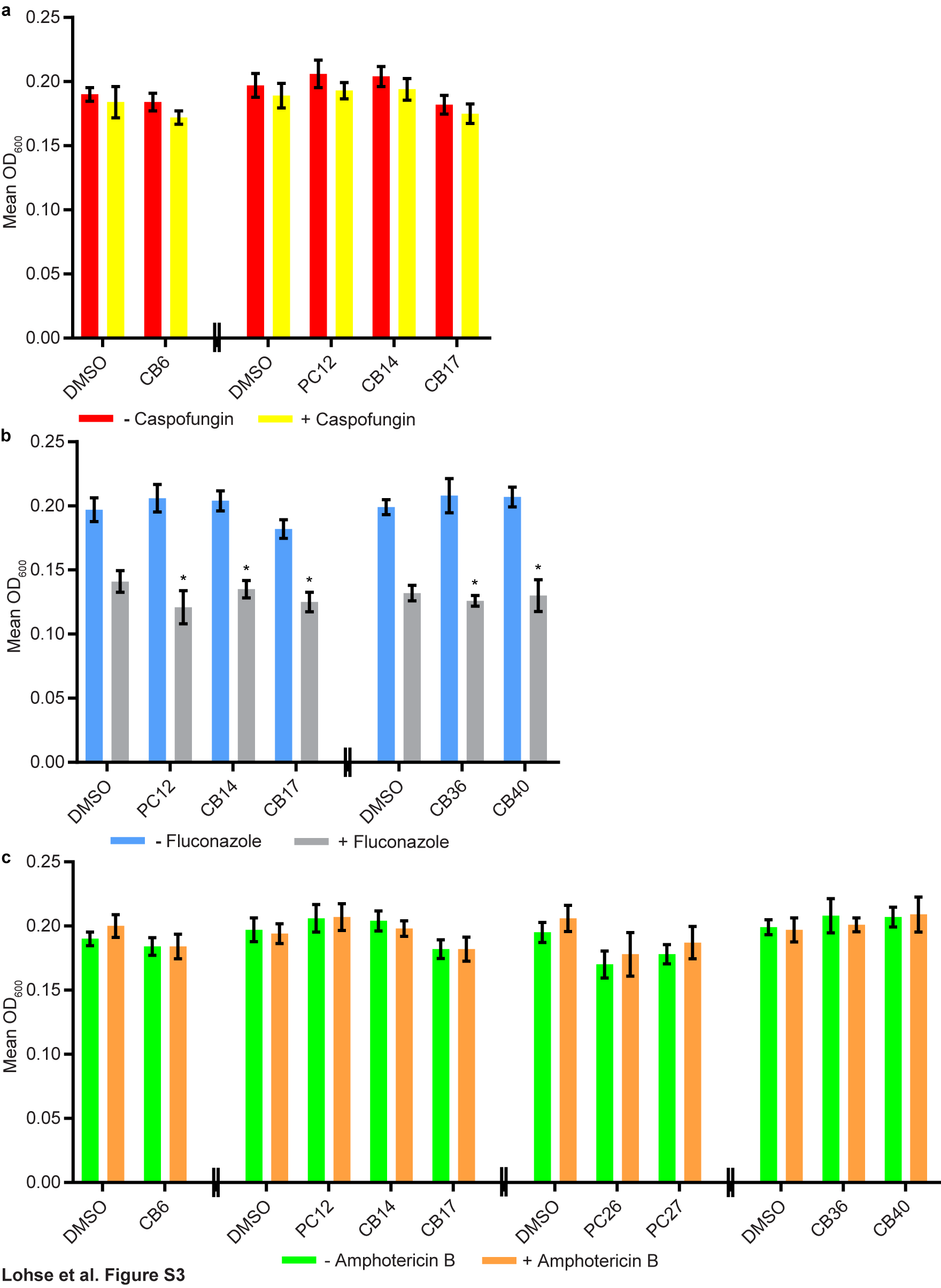

Supplement: Supplementary file 1 [file jof-07-00009-s001.zip › Lohse et al Figure S3.tif]
